# Promoting employee wellbeing and preventing non-clinical mental health problems in the workplace: a preparatory consultation survey

**DOI:** 10.1186/s12995-023-00378-2

**Published:** 2023-08-15

**Authors:** Evelien Coppens, Bridget Hogg, Birgit A. Greiner, Charlotte Paterson, Lars de Winter, Sharna Mathieu, Johanna Cresswell-Smith, Birgit Aust, Caleb Leduc, Chantal Van Audenhove, Arlinda C. Pashoja, Dooyoung Kim, Hanna Reich, Naim Fanaj, Arilda Dushaj, Katherine Thomson, Cliodhna O’Connor, Ana Moreno-Alcázar, Benedikt L. Amann, Ella Arensman

**Affiliations:** 1 LUCAS Center for care research and consultancy, KU Leuven, Leuven, Belgium; 2 Centre Fòrum Research Unit, Hospital del Mar Research Institute, Barcelona, Spain; 3 Centro de Investigación Biomédica en Red de Salud Mental (CIBERSAM), Instituto Carlos III, Madrid, Spain; 4 School of Public Health, University College Cork, Cork, Ireland; 5 Nursing, Midwifery and Allied Health Professionals Research Unit (NMAHP-RU), University of Stirling, Stirling, Scotland; 6 Phrenos Center of Expertise, Utrecht, the Netherlands; 7 Australian Institute for Suicide Research and Prevention & W.H.O Collaborating Centre for Research and Training in Suicide Prevention, School of Applied Psychology, Griffith University, Queensland, Australia; 8 Finnish Institute for Health and Welfare, Helsinki, Finland; 9 National Research Centre for the Working Environment, Copenhagen, Denmark; 10 National Suicide Research Foundation, Cork, Ireland; 11 School of Hygiene and Tropical Medicine, Population Health, Global Public Health, Public Health England, LondonLondon, UK; 12 European Alliance Against Depression E.V, Leipzig, Germany; 13 Depression Research Centre, German Depression Foundation, Leipzig, Germany; 14 Per Mendje Te Shendoshe, Prizren, Kosovo; 15 Alma Mater Europaea Campus Rezonanca, Prishtina, Kosovo; 16 Community Centre for Health and Wellbeing, Tirana, Albania; 17 International Association for Suicide Prevention, Washington, DC USA; 18 Institute of Mental Health, Hospital del Mar Barcelona, Barcelona, Spain; 19 Department of Psychiatry and Psychotherapy, Klinikum Der Universität München, Munich, Germany; 20 Universitat Pompeu Fabra, Barcelona, Spain; 21 School of Public Health and National Suicide Research Foundation, University College Cork, Cork, Ireland; 22 Australian Institute for Suicide Research and Prevention, Griffith University, Brisbane, Australia

**Keywords:** Barriers and facilitators, Construction, Gender, Healthcare, Information and communication technologies (ICT), Mental health, Mental health promotion and intervention in occupational settings (MENTUPP), Small and medium enterprises

## Abstract

**Background:**

Small and medium-sized enterprises (SMEs) face major financial losses due to mental health issues affecting employees at all levels but seldom apply programs to promote wellbeing and prevent mental health issues among employees. To support the development of a multi-country workplace-based mental health intervention for SMEs (MENTUPP), a multinational consultation study was conducted. The study aimed to examine the experiences and needs of SMEs concerning the promotion of employee wellbeing, and the prevention and management of non-clinical mental health problems in workplaces.

**Methods:**

A survey consisting of open and closed questions was designed to assess key informants’ opinion about the acceptability, the use, and the implementation of interventions to promote wellbeing and prevent mental health issues in the workplace. Academic experts and representatives of SME organisations, specific sector organisations, labour or advocacy groups, and occupational health organisations across the nine MENTUPP intervention countries (eight European countries and Australia) were invited to complete the survey. Data were collected via the online platform Qualtrics. Sixty-five of 146 informants responded, representing a 44.5% response rate. Descriptive statistics were used to analyse the quantitative data and qualitative data were analysed through thematic analysis.

**Results:**

Measures to create mentally healthy workplaces were most used in SMEs, while more specific mental health interventions, such as training staff on how to promote wellbeing, were hardly used. Managers lack resources to implement mental health interventions and are concerned about employees spending too much time on these interventions during working hours. Receiving information about the economic benefits of mental health interventions and hearing successful testimonials from other SMEs can persuade managers otherwise. Employees have concerns about confidentiality, discrimination and stigma, and career opportunities when using such interventions.

**Conclusions:**

The study identifies a variety of challenges, needs and possibilities related to implementing mental health interventions in SMEs. Employers need to be convinced that investing in mental health in the workplace is worth their time and money. This requires more studies on the (cost-)effectiveness of mental health interventions. Once employers are engaged, their knowledge and competencies about how to implement such interventions should be increased and privacy concerns of employees to participate in them should be addressed.

**Supplementary Information:**

The online version contains supplementary material available at 10.1186/s12995-023-00378-2.

## Background

Periods of high stress or upsetting events, such as stressful working conditions, job loss, the death of a beloved one, or a divorce, can lead to chronic stress and reduced wellbeing. Without receiving support, these problems may be one of the factors implicated in the onset and maintenance of clinical mental health problems, such as major depressive disorder or generalised anxiety disorder, causing a major impact on one’s personal life and the wider society [27]. Workplace stress can affect people when they are under high pressure and have demands at work that are beyond their capacities and coping mechanisms [44, 46]. In the long run, when this work-related stress is not managed, it can result in burnout which is characterized by exhaustion, negative feelings towards one's job, and lower efficacy at work (i.e. decreased productivity, retention, absenteeism and presenteeism) [29, 44]. Although clear cut burnout prevalence estimates are lacking, the World Health Organisation (WHO) acknowledges that this syndrome has an enormous negative impact on the work and personal life of workers, affecting the public health and economy of many Western countries [16, 23]. The COVID-19 pandemic further increased the psychological burden on workers, making the importance of health at work more important than ever [53].

Studies demonstrate a strong link between work and mental health and emphasize the importance of developing and implementing interventions to create more mentally healthy workplaces [12, 39]. Work can be the direct cause of mental health issues: a poor psychosocial work environment, characterized by low job control, high job demands, job insecurity and effort-reward imbalance, may have adverse effects on mental wellbeing and can increase the risk of mental health problems [33, 41, 46, 48]. Additional risk factors disproportionately affect women, such as work-family conflict [4], and workplace sexual harassment [47], while those who do not conform to standard gender stereotypes are at additional risk of bullying [43]. Contrary, work environments that reduce work-related risk factors and promote the positive aspects of work can contribute to employees’ mental health, making them feel more satisfied, engaged and productive, which in turn predicts higher organizational performance and productivity [12, 51]. Thus, reduced psychosocial risks in the workplace can positively impact mental health. However, workplace-based interventions can also play an important role in supporting people to improve their mental health even when the workplace is not one of its causes. Currently, a large proportion of people with mental illness do not seek help, due to a range of factors including not recognising symptoms and stigma related to mental illness [19, 50]. Supporting these individuals to seek help can reduce mental health-related business costs and improve outcomes [24]. Interventions that aim to address mental health problems in the workplace by improving mental health literacy, strengthening skills for early intervention, promoting help-seeking behaviour, and facilitating return-to-work after a mental illness-related absence, appear very promising [39]. At the same time, it is important to tackle stigmatizing attitudes, as such attitudes among peers often prevent people from disclosing their problems and seeking help [27, 35].

Today, mental health interventions are increasingly being used in large-sized organizations whereas small-to-medium sized enterprises (SMEs), which account for 94% of all enterprises in the European Union, hardly implement such interventions [30]. Commonly cited reasons for this are a lack of interest and resources, a lack of priority of the business, a lack of awareness of their responsibilities, and limited knowledge and competencies to adopt and integrate such programs [24, 36]. Furthermore, there is a lack of psychosocial interventions tailored to the specific needs of SMEs. The MENTUPP project (mental health promotion and intervention in occupational settings) is a large-scale EU-funded project aiming to address this gap (https://www.mentuppproject.eu/). The project's objective is to develop, implement and evaluate an evidence-based multilevel intervention targeting both non-clinical (stress, burnout, wellbeing, depressive symptoms) and clinical (depressive, anxiety disorders) mental health problems, and combating stigmatizing attitudes that are often associated with mental ill health [1], in employees of SMEs in the sectors of construction, healthcare, and Information and Communications Technology (ICT). The three sectors were chosen because the risk of mental health problems is particularly pronounced in these sectors. In construction, employees face common psychosocial stressors such as short-term contracts, job uncertainty, and mental overload due to high quality standards, job performance ratings, and executing complex and routine tasks [42]. In healthcare, employees are regularly confronted with the stress and suffering of patients, but are also subject to long working hours, understaffing, and excessive work pressure [37], contributing to high rates of burnout and occupational stress in the healthcare sector [52]. Furthermore, healthcare workers suffered a particularly strong exposure to psychosocial risk factors during the COVID-19 pandemic [2], which coincided with the timing of this survey. Suicide rates are much higher than the general population in construction and healthcare workers [38]. ICT is a relatively new and fast-growing sector characterized by time pressure, work interruptions, multi-tasking and work-life imbalance, leading to stress, anxiety, burnout and worse self-reported health outcomes [15]. Furthermore, the ICT sector exemplifies the digitalisation which is happening across many sectors, which create significant occupational safety and health challenges, with for example digital technologies controlling the pace of work, and risks including increased workload, surveillance, and lowered autonomy [25]. Nevertheless, the aforementioned characteristics require some nuance, as there is a diversity of job types and required skills within each sector.

Currently, little is known about which mental health interventions are commonly used in SMEs. Also, evidence about the effectiveness, the perceived acceptability and the appropriateness of these interventions in SMEs is scarce [21]. To support the development of the evidence-based MENTUPP intervention, a consultation survey was conducted to complement the identified gap in the scientific literature. When no evidence is available, consultation surveys are a recommendable approach [34]. For this survey, we required informants to have expertise based on a minimum of five years’ experience in a role which requires knowledge of mental health interventions in an organizational context. The range of informants was diverse to capture not just academic viewpoints, but also viewpoints from leaders in SMEs and in the construction, health, and ICT industry, specialists in occupational health, and the viewpoints of employees from labour union representatives, and the viewpoint of people with a lived experience of mental illness through advocacy organisations. The full survey covers a broad range of topics to underpin the development of the multiple levels of the MENTUPP intervention. Results regarding mental illness, related stigma and the impact of COVID-19 are reported elsewhere [22]. This article addresses the following research questions related to wellbeing and non-clinical mental health problems in the workplace:

1. What is the acceptability of interventions to promote wellbeing and prevent mental health issues in the workplace?
2. What interventions are commonly used to promote mental health in the workplace and perceived as a good practice?
3. What are important barriers and facilitators to consider when implementing mental health interventions in the workplace?
4. What needs do workplaces and SMEs have in order to improve their activities on promoting mental health and preventing mild mental health difficulties?
5. What gender specific needs exists and how should these be addressed in the workplace?

## Method

### Materials

A comprehensive semi-structured bespoke survey was designed to assess key informants’ views on the five general research questions relating to wellbeing and non-clinical mental health problems in the workplace. In addition, the survey included a few questions asking about the informants’ sociodemographic background. A mix of closed and open questions were formulated by researchers from the MENTUPP consortium to obtain both quantitative and qualitative data. The questions were formulated to address knowledge gaps in the extant literature in order to understand how best to provide an intervention for the sectors of construction, ICT, and healthcare in an SME context, at both supervisor and employee level. Existing data was used as the basis for the questions; for example, a question on commonly used interventions to promote wellbeing was based on recent research showing the need for an integrated approach with strategies at different levels [27, 35, 39] thereby the question was formulated to understand to what extent the experts assess that this best practise is currently in place. The questions were iteratively reviewed by the MENTUPP consortium in consultation with external experts until consensus was achieved.

Table 1 specifies the survey items and their respective response categories. Four-point (i.e. not all, to a small extent, somewhat, to a large extent) or five-point (i.e. strongly disagree, disagree, neutral, agree, strongly agree) Likert-type scales were used to answer the closed-ended questions, with most items containing a "don't know" option in addition to the standard answer options. This last option was included to avoid participants answering items outside their realm of expertise and thus introducing bias. For the open-ended questions, participants had to write their answers in their own words.

**Table 1:** Survey items

Survey topics	Items	Response
Acceptability of interventions	To what extent do you think that managers/supervisors might have the following concerns when it comes to implementing mental health interventions within the workplace?	Four-point scale: 1 = not at all, 2 = to a small extent, 3 = somewhat, 4 = to a large extent Optout option: don’t know
	1. Thinking that the workplace is not responsible for employees’ mental health	
	2. Thinking that staff will hesitate to participate in interventions in the workplace	
	3. Concern about lack of resources for implementation	
	4. Concern about employees accessing interventions during work time or using work resources	
	5. The workplace is not the appropriate setting for such interventions	
	To what extent do you think that the following arguments may influence managers/supervisors when deciding whether or not to implement mental health interventions within the workplace:	Four-point scale: 1 = not at all, 2 = to a small extent, 3 = somewhat, 4 = to a large extent Optout option: don’t know
	1. Information on the economic benefits it could bring to the workplace	
	2. Information on the social benefits it could bring to the workplace	
	3. Testimonials from managers/supervisors who have implemented mental health interventions and noted positive changes within the business	
	4. Scientific research on the benefits of mental health interventions	
	5. Simple implementation which requires minimal manager/HR time	
	6. Minimal requirement of employee time	
	To what extent do you think that the following issues may prevent an employee from participating in mental health interventions within the workplace setting?	Four-point scale: 1 = not at all, 2 = to a small extent, 3 = somewhat, 4 = to a large extent Optout option: don’t know
	1. Concerns about confidentiality	
	2. Concerns about discrimination/stigma	
	3. Concerns about career progression/job security	
	4. Thinking that the workplace should not get involved when employees have mental health problems	
	Please rate the following statements about accessing tools online in terms of agreement:	Five-point scale: 1 = strongly disagree, 2 = disagree, 3 = neither agree nor disagree, 4 = agree, 5 = strongly agree
	1. Employees may feel uncomfortable accessing online mental health interventions while being at work	
	2. Accessing an online intervention while in the workplace could have negative repercussions for the employee	
	3. Employees accessing an online intervention through the workplace could have negative repercussions for the employers/business/SME	
	4. Employees in the area have easy access to a computer during working hours	
	5. It would be easier for employees to access an intervention through their personal smartphone	
Commonly used interventions	To what extent does the average workplace:	Four-point scale: 1 = not at all, 2 = to a small extent, 3 = somewhat, 4 = to a large extent Optout option: don’t know
	1. Create mentally healthy workplaces by e.g. providing flexible and supportive working conditions and/or avoiding stressful working conditions, such as long working hours, excessive workload or poor supervisory support	
	2. Have a strategic and coordinated organizational approach to promote employees' mental wellbeing	
	3. Carry out needs assessments among employees to inform an organizational approach to promote mental wellbeing	
	4. Provide training for managerial/HR staff on promoting wellbeing in the workplace	
	5. Provide psychological support services to employees (e.g. counselling, stress management)	
	6. Have a strategic and coordinated organizational approach to reduce stigma related to mental health problems	
Barriers and facilitators to implement interventions	Can you suggest up to five key barriers that you are aware of when implementing interventions aimed at promoting employee mental health?	Open text
	Can you suggest up to five key facilitators that you are aware of which have helped when implementing interventions aimed at promoting employee mental health?	Open text
Needs of workplaces	To what extent do you think that workplaces need more information about:	Four-point scale: 1 = not at all, 2 = to a small extent, 3 = somewhat, 4 = to a large extent Optout option: don’t know
	1. How to create mentally healthy working conditions	
	2. Factors contributing to work stress and burnout 3. How to establish policies about creating mentally healthy workplaces 4. How to carry out a needs assessment to inform an organizational approach to promoting wellbeing	
	5. How to strengthen people management skills among senior staff/HR staff in order to detect and handle mental health problems	
Gender specific needs	What gender-specific aspects exist in terms of help-seeking behaviour related to mental health issues?	Open text
	What gender-specific aspects should be considered when supporting an employee’s mental health?	Open text
	What gender-specific aspects need to be considered in male dominated and female dominated workplaces in terms of creating a mentally healthy workplace?	Open text

### Recruitment and participants

A broad group of key informants was invited to take part in the survey, including: (1) academics with expertise in workplace mental health promotion and occupational health; (2) steering group members or directors of SME organisations; (3) steering committee members and representatives of the construction, healthcare or ICT sector associations; and (4) occupational health specialists and representatives of associations, labour unions and advocacy groups. The range of specialist areas was chosen to gather not just academic data but also an understanding of what people representing industry leaders, employees, and those with lived experience of mental illness, assessed the MENTUPP intervention needed to provide. The following exclusion criteria were used: (1) less than 5 years’ experience in their domain; (2) being a member of the MENTUPP consortium; and (3) being < 18 years old. Informants were recruited from the nine countries where the MENTUPP intervention will be trialled: Albania, Australia, Finland, Germany, Hungary, Ireland, Kosovo, the Netherlands, and Spain. The selection of countries provides a culturally and economically diverse range of contexts for trialling the MENTUPP intervention, thereby giving added strength to the project. Inclusion of experts from all the MENTUPP countries was necessary to understand if there were specific factors to take into account to ensure a successful intervention in each geographical area. Different strategies were used to identify participants for this survey, including networking (i.e. via recommendation by members of the local MENTUPP steering groups and experts in the field), identification through organization websites, snowballing, and internet searches. In each country, the lead researcher was asked to identify 5 to 25 key informants (see Table 2 for the number of invited informants per country).

**Table 2:** Number of invited respondents, number of respondents, and sample distribution per country and field of expertise

			**Field of expertise**				
**Country**	**Invited**	**Responded**	SMEs in construction, health or ICT	Academic field	Organisations providing services for SMEs or groups of SMEs	Occupational health specialist, labour unions or advocacy groups	Other
Albania	25	16	8	4	0	1	3
Australia	14	2	1	1	0	0	0
Finland	15	6	0	3	0	1	2
Germany	15	4	4	0	0	0	0
Hungary	20	10	5	0	3	0	2
Ireland	6	3	0	2	0	1	0
Kosovo	22	10	6	0	1	0	1
Netherlands	12	7	3	2	0	1	1
Spain	17	9	5	3	0	1	0
**Total**	146	65	32	15	4	5	9

### Procedure

The survey was sent out by the lead researcher within every country to the 146 identified informants between 15/09/2020 and 5/10/2020. Two to three reminders were sent to encourage participants to complete the survey in time. Participation was voluntary and only proceeded following informed consent. Survey completion was estimated to take approximately 20 to 50 min. The survey and informed consent document were prepared in English and local language versions were additionally provided for Albania, Germany, Hungary, Kosovo, the Netherlands, and Spain. The English versions of the informed consent document and survey were uploaded into Qualtrics, which is a GDPR-compliant software for conducting online surveys (version 21 of Qualtrics, www.qualtrics.com). Experts in Australia, Finland, and Ireland, and experts from other countries who spoke fluent English, filled out the survey directly in Qualtrics. Local language versions were administered via Word or paper format, and the results were translated into English and entered into Qualtrics by each country’s lead researcher.

### Analyses

Survey responses in relation to the closed questions were analysed using descriptive statistics. For each item, the percentages were calculated. Additionally, the median response (Mdn) and the interquartile range (IQR; the distance between the 25th and the 75th percentiles) were calculated to determine the levels of agreement on the items, using the ordinal data from the Likert scales with the category “I don’t know” omitted. The answers on the open-text questions were analysed by two independent researchers with mutual consensus relying on a reflexive thematic analysis approach in which the coding process occurred in an organic and subjective manner requiring a deep understanding of the data and reflexive thinking of the researchers [6]. The reflexive approach consists of six steps: familiarisation with the data; reflexive coding of the data; generating initial themes; reviewing and developing themes; refining, defining and naming themes; and writing up. The results report for each theme the number of different comments made by the participants and for each comment the number of participants who suggested it.

## Results

### Characteristics of participants

A total of 146 informants across the nine countries of interest were invited to take part in the consultation survey. Of these, 65 individuals (26 female, 37 male, and 2 other) participated, corresponding to a response rate of 44.5%. Three quarters of the participants were older than 40 years and two-thirds of the participants had over 10 years of experience in their field. Table 2 presents the number of participating informants per country and their field of expertise. To provide a context for the results over different geographical areas, in Supplementary Table 1 can be seen general information per country regarding the percentage of the workforce in SMEs and in each sector (construction, ICT, and healthcare), which shows that across all MENTUPP countries, SMEs makeup over 99.5% of companies, although in Germany, Hungary, and Ireland, they employ less than half of the overall workforce. The construction sector accounts for the largest share of the workforce in Albania and Kosovo (9.0% and 9.5% respectively), while it accounted for less than 6% of the workforce in Germany and the Netherlands, while Finland has the largest proportion of workers in both the healthcare and ICT sector of the countries where data is available. Furthermore, in Supplementary Table 2 can be seen data from the European Survey of Enterprises on New and Emerging Risks (ESENER) survey (2019) regarding the extent to which psychosocial risks are addressed in each EU country, showing that Finland has the greatest percentage of companies with measures to address psychosocial risks in place. In our survey, although all countries were represented in the responses, there was a considerable variability in the number of participants per country (see Table 2) and the response rate differed across countries, *X*^*2*^(8) = 57.5, *p* < 0.01, with high response rates in Albania (64%) and Spain (75%), intermediate rates in Finland (40%), Hungary (50%), Ireland (50%), Kosovo (36%) and the Netherlands (41%), and low rates in Australia (14%) and Germany (27%). Almost half of the participants represented the construction, health or ICT sectors, whereas about a quarter were academics. The informants’ field of expertise was equally distributed across countries, *X*^*2*^(32) = 41.3, *p* = 0.13.

### Acceptability of mental health interventions

Table 3 depicts the participants’ responses on items assessing the acceptability of mental health interventions in the workplace which was rated on a four-point Likert scale. Half of the informants believed that a lack of resources (52.3%) and the use of mental health interventions during work time (49.2%) may largely prevent managers from implementing them.

**Table 3:** Participants’ responses on items assessing the acceptability of mental health interventions in the workplace (percentages, Mdn, IQR)

	**Not at all**	**To a small extent**	**Somewhat**	**To a large extent**	**Don’t know**	**Mdn**	**IQR**
**Possible concerns of managers to implement such interventions:**							
Workplace is not responsible	6.2	15.4	43.1	33.8	1.5	3	1
Staff will hesitate to participate	3.1	10.8	55.4	27.7	3.1	3	1
Lack of resources	1.5	10.8	33.8	52.3	0	4	1
Use interventions during work time	0	13.8	32.3	49.2	3.2	4	1
Workplace is an inappropriate setting	6.2	10.8	55.4	26.2	0	3	1
**Arguments that may convince managers to implement such interventions:**							
Information on economic benefit	3.1	7.7	20	66.2	1.5	4	1
Information on social benefits	1.5	21.5	33.8	38.5	1.5	3	1
Successful testimonials from other managers	0	9.2	21.5	61.5	6.2	4	1
Scientific research on benefits	6.2	23.1	40	24.6	4.6	3	2
Minimal time investment for managers	1.5	10.8	38.5	46.2	1.5	3	1
Minimal time investment for employees	4.6	6.2	43.1	41.5	3.1	3	1
**Concerns of employees to use such interventions:**							
Confidentiality	0	3.1	26.2	69.2	1.5	4	1
Discrimination and stigma	0	4.6	26.2	69.2	0	4	1
Career progression and job security	0	9.2	18.5	70.8	0	4	1
Workplace should not be involved in mental wellbeing	4.6	16.9	50.8	24.6	0	3	1

According to the majority of informants, receiving information about the economic benefits of mental health interventions (66.2%) and hearing testimonials from other managers who have successfully implemented such interventions (61.5%) may convince managers to a large extent to implement such interventions. In addition, more than 40 percent of informants believed that interventions requiring minimal time investment from managers (46.2%) and employees (41.5%), can significantly increase managers’ motivation to implement mental health interventions.

Concerns that may largely dissuade employees from using mental health interventions at work are, according to the majority of informants, concerns about confidentiality (69.2%), discrimination and stigma (69.2%), and career progression and job security (70.8%).

Table 4 outlines the informants’ responses on items assessing the acceptability of online mental health tools in the workplace which were rated on a five-point Likert scale. Two-thirds of informants agreed that employees may feel uncomfortable using online interventions in the work environment (67.7%). In addition, almost half of informants agreed that employees may have easy access to a computer during working hours (53.9%) and the majority of informants agreed that employees may have easier access to online interventions through their personal smartphone (69.2%).

**Table 4:** Participants’ responses on items assessing the acceptability of online tools in the workplace (percentage, Mdn, IQR)

	**Strongly disagree**	**Disagree**	**Neutral**	**Agree**	**Strongly agree**	**Mdn**	**IQR**
**Accessing online tools while being at work:**							
Is uncomfortable	3.1	10.8	18.5	44.6	23.1	4	1
May have negative repercussions for employees	6.2	26.2	24.6	29.2	13.8	3	2
May have negative repercussions for organizations	12.3	24.6	33.8	21.5	7.7	3	2
**Employees have:**							
Easy access to a computer during working hours	3.1	9.2	33.8	30.8	23.1	4	1
Easier access to a smartphone	0	6.2	24.6	36.9	32.3	4	2

### Commonly used mental health interventions

Table 5 provides an overview of the informants’ responses on items assessing commonly used interventions to promote mental wellbeing in the workplace which had to be rated on a four-point Likert scale. The intervention measures to create a mentally healthy workplace was rated by the informants as the most commonly used mental health intervention in organizations, with 40% of informants indicating that it is somewhat used and 13.8% of informants indicating that it is used to a large extent.

**Table 5:** Participants’ responses on items examining commonly used interventions to promote mental wellbeing in the workplace (percentages, Mdn and IQR)

Commonly used interventions in the workplace	Not at all	To a small extent	Somewhat	To a large extent	Don’t know	Mdn	IQR
Measures to create a mentally healthy workplace	9.2	35.4	40	13.8	1.5	3	1
A strategic and coordinated approach to promote employees' mental wellbeing	18.5	52.3	18.5	7.7	3.1	2	1
Needs assessments among employees to inform an organisational approach to promote mental wellbeing	1.5	35.4	16.9	7.7	38.5	2	1
Training for managers and human resources staff on promoting wellbeing	21.5	35.4	27.7	6.2	9.2	2	1
Psychological support services to employees	26.2	32.3	24.6	12.3	4.6	2	2
A strategic and coordinated approach to reduce stigma	50.8	26.2	13.8	6.2	3.1	1	1

A strategic and coordinated approach to promote employees' mental wellbeing is, according to most informants, not a common practice in organizations, with 70.8% of informants believing that this approach is used to a small extent or not all. The same is true for a strategic and coordinated approach to reduce stigma, with 77% of informants assessing that such interventions are used to a small extent or not at all.

### Barriers and facilitators to implement mental health interventions

In an open text question, informants identified barriers that may hinder engagement in and implementation of mental health interventions. At the organizational level, mental health not being considered as a priority in SMEs (*n* = 19/65; 29%) (e.g. *“lack of interest in companies, insufficient understanding of the importance of mental health at work, mental health as the last priority on the list”*), financial or budgetary issues (*n* = 13/65; 20%) (e.g. *“financial implications, budgetary issues, fear of costs without benefits”*), staff having insufficient knowledge about mental health policies and interventions (*n* = 11/65; 17%) (e.g. *“lack of knowledge at the management level, being unable to link mental health to the organisations’ strategy and business goals”*), time management problems (*n* = 11/65; 17%) (e.g. *“lack of time, personnel having too much other work to do, unrealistic timelines putting new initiatives under pressure”*), lack of structures and competent professionals within the organization to provide mental health interventions (*n* = 11/65; 17%) (e.g. *“no health policy integrated in the overall company policy, lack of human resources to intervene in difficult situations, lack of psychological support structures in the organisation”*), and a lack of commitment from managers (*n* = 10/65; 15%) (e.g. *“lack of leadership commitment, no support from the management or the board, lack of managerial will”*) were cited by the informants as the main barriers that may discourage SMEs from implementing mental health interventions. At the individual level, the fear of stigmatizing attitudes and prejudices among colleagues (*n* = 24/65; 36.9%) (e.g. *“being ashamed to talk about mental difficulties, cultural stigma towards mental health problems, fear of what others will say”*), problems related to discretion, confidentiality and mistrust (*n* = 12/65; 18%) (e.g. *“lack of discretion about employees’ mental health, confidentiality issues, distrust in the whole process”*), the fear of negative effects on the career (e.g., job loss, loss of status, etc.) (*n* = 11/65; 17%) (e.g. *“fear of being fired, opening up about mental health issues will have a negative impact on the career, fear of losing status and being relegated”*), and general disinterest (*n* = 10/65; 15%), (e.g. *“irrelevant for working population, lack of motivation among employees, it’s being perceived as something with little utility”*) were often reported by informants as possible barriers that may discourage employees from participating in mental health interventions.

The informants mentioned a few facilitators at the organizational level that may promote the implementation of mental health interventions in SMEs such as: strengthening the commitment of managers/supervisors (*n* = 14/65; 22%) (e.g. *“increasing the commitment of managers, highlighting the economic imperative of a mentally healthy workforce, increase employer support”*) and arranging regular communications about mental health (e.g. fixed item during weekly meetings, talks initiated by gatekeepers or HR) (*n* = 11/65; 17%) (e.g. *“regular collective and individual meetings of direct managers with employees, gatekeepers in the workplace as a key pillar, group meetings to share ideas and experiences”)*. At the individual level, employees could be encouraged to participate in mental health interventions among others by: raising their interest through media campaigns (*n* = 23/65; 35%) (e.g. *“raise awareness *via* media, gain acceptance *via* literature”*), strengthening personal relationships between employees to reduce stigmatizing attitudes and creating the opportunity to talk about mental health issues (*n* = 13/65; 20%) (e.g. *“install a buddy system, organise social events in the company, invest in personal relationships”*), enabling flexibility in the workplace where needed (i.e. working hours and adaptation of tasks) (*n* = 11/65; 17%) (e.g. *“allow flexible working hours, install the option to work remotely, provide support to handle daily life challenges”*), and increasing employees’ knowledge on mental health (e.g. through scientific literature, education and facts) (*n* = 11/65; 17%) (e.g. “*organise mindfulness seminars, share facts, evidence and local information”*).

### Needs of workplaces

Table 6 provides an overview of the informants’ responses on the items examining the type of information, tools, and advice that would be helpful for organizations, which were rated on a four-point Likert scale. At least half of the informants indicated that there is a great need for information, tools and advice on the following four topics: how to create mentally healthy working conditions (58.5%), what factors contribute to work stress and burnout (53.8%), how to establish policies about creating mentally healthy workplaces (50.8%), and how to strengthen people management skills among senior/HR staff to detect and handle mental health problems (50.8%).

**Table 6:** Participants’ responses on items examining the type of information, tools, and advice that would be helpful for organizations (percentages, Mdn, IQR)

Needs	Not at all	To a small extent	Somewhat	To a large extent	Don’t know	Mdn	IQR
**Information, tools and advice on:**							
How to create mentally healthy working conditions	1.5	4.6	33.8	58.5	1.5	4	1
Factors contributing to work stress and burnout	1.5	15.4	27.7	53.8	1.5	4	1
How to establish policies about creating mentally healthy workplaces	0	10.8	35.4	50.8	3.1	4	1
How to carry out a needs assessment to inform an organisational approach to promoting wellbeing	0	20	41.5	33.8	4.6	3	1
How to strengthen people management skills among senior/human resources staff in order to detect and handle mental health problems	1.5	13.8	33.8	50.8	0	4	1

### Gender-specific needs

Results regarding gender-specific needs were assessed very differently by the 65 informants. In response to the three open text questions, 34 informants (52%) highlighted a large gender difference in terms of help-seeking behaviour, with women being viewed as more likely to ask for help than men (e.g. *“males tend not to talk much about mental health issues in their workplace and therefore are not able to ask for help while females tend to ask more often for help; men try to hide their problems or try to take care of themselves for example with alcohol”*), whereas nine informants (13.8%) asserted that there is no gender difference (e.g. *“there is a gender difference in help-seeking; it is not an issue of gender rather a matter of holistic thinking; there is no gender difference in help-seeking in the big city, in the countryside perhaps there is"*). Moreover, 17 informants (26%) confirmed that it is important to address gender-specific needs when supporting employees’ mental health (e.g. *“male language should be used in this field and it should be learned how to reach men with relatively traditional masculine identity better; men see support as a sign of weakness and lack of strength which is very disturbing for them and requires additional attention; there is a need for gender specific approaches and understandings around mental health and the support that should be offered”*), while 11 informants (17%) argued that mental health support is important for everyone regardless of gender and hence should be gender-neutral (e.g. *“there should be gender neutrality in all communication and facilities; we must insist that mental health is important for all people regardless of gender; health and mental health are human conditions so gender should not be an issue in mental health support”)*. Finally, 24 informants (37%) agreed that gender-specific needs should be considered when aiming to create a mentally healthy environment in male-dominated and female-dominated workplaces (e.g. *“different specifics require different attention, the genders that are in minority (especially non-binary genders) should get support to feel accepted and welcomed in the community; workplaces that are dominated by women, should consider family reconciliation, wage inequality and work overload”*), whereas five informants (7.7%) felt that it is important to step aside from feminine and masculine roles and to consider instead the mental health needs of people in general regardless of gender when promoting mental wellbeing in the workplace (e.g. *“being gender-specific is not a constructive approach, instead we should focus on making work places better in general; I am totally against these feminism and masculism roles, there are both dominant males and females who can be savage, so it is better to treat people in function of what they need and not in function of the gender to which they belong”*).

## Discussion

A study led by the WHO estimates that mental health difficulties cost the global economy US$ 1 trillion every year in lost productivity [53]. These costs are expected to rise as the COVID-19 pandemic has aggravated financial instability, prompted restructuring, and accelerated the pace of change in enterprises, which may create new psychosocial risks or exacerbated existing ones [53]. Workplaces that promote mental health and support people with mental disorders are according to the WHO more likely to reduce absenteeism, increase productivity and benefit from associated economic gains, but there is a need to support SMEs more thoroughly in implementing such measures [24, 36]. The current survey assessed key informants’ opinion about the use and acceptability of interventions to promote wellbeing and prevent mental health issues in SMEs, reasons for not using such interventions by SMEs, and gender specific needs to watch out for. Our study included a diverse set of key informants from a wide range of countries, and a key finding was the strong level of agreement regarding the need for psychosocial interventions in an SME context. This data supports the feasibility and utility of developing an intervention such as the MENTUPP intervention to be used across a wide range of contexts, as a universal intervention for SME employees from the sectors of construction, healthcare and ICT.

Measures to create mentally healthy workplaces are according to our results the most commonly used mental health intervention in SMEs. Such measures include providing flexible and supportive working conditions and/or acting to avoid stressful working conditions, such as long working hours, excessive workload or poor supervisory support. More specific mental health interventions such as training managers or human resources staff on how to promote wellbeing, providing psychological support services to employees, conducting a needs assessment to inform an organizational approach, developing and implementing a strategic and coordinated approach to promote employees' mental wellbeing, and developing and implementing a strategic and coordinated approach to reduce stigma are according to the informants in our study used little or not at all in SMEs. The ESENER survey, a cross-national survey that examines how European SMEs manage safety and health risks in practice (EU-OHS 2020), similarly reports that roughly half of the participating SMEs are putting in place measures to address psychosocial risks and create a healthier workplace. Examples of these measures are allowing employees to take more decisions on how to do their job, arranging confidential counselling for employees, organizing training on conflict resolution, and reorganizing work to reduce job demands and work pressure (EU-OHS 2020). Benning et al. [3] also report that in Dutch SMEs the most frequently used preventive health measures focus on improving working conditions, whereas interventions that aim to promote healthier lifestyles (e.g. sports/fitness subscriptions, lifestyle counselling, stress-management courses) are hardly used.

Our results show that there are several barriers at the level of the organisation that prevent SMEs from implementing interventions to promote employee wellbeing. According to nearly a third of our informants, investing in employees’ mental health is not a priority and therefore usually not part of the organization’s culture. Financial or budgetary issues as well as time management problems are also frequently mentioned as barriers by the informants. Furthermore, several informants pointed out that SMEs lack knowledge about mental health policies and interventions, and lack structures and competency to implement such interventions within the organisation, which may affect supervisors’ commitment to put their shoulders to the wheel. A literature review conducted by McCoy et al. [30] examined evidence regarding the adoption and efficacy of worksite health promotion programs in small businesses and identified comparable barriers. The direct and indirect costs of adopting a program was perceived as a major hindrance for small businesses, as they have fewer resources to invest in health programs that may or may not pay off and cannot afford to hire a staff person who is responsible for implementing such programs. McCoy et al. [30] reported two additional barriers that were not mentioned in our study: first, especially in small businesses it can be hard to justify targeted interventions that may only reach a small number of employees; second, companies do not have time and expertise to evaluate the efficacy of such interventions which is important to decide about its sustainability. The barriers mentioned by our informants are also similar to the findings reported by Benning et al. [3], who recently investigated determinants for implementing measures to prevent musculoskeletal and mental disorders in Dutch SMEs. The identified determinants relate to 10 distinct themes: (1) available resources (both finances and staff) (2) complexity of implementation of measures, (3) awareness, (4) knowledge and expertise, (5) availability of time, (6) employer and worker commitment, (7) workers’ openness for measures, (8) communication, (9) workers’ trust and autonomy, and (10) integration in organizational policy.

Except for barriers at the level of the organisation, our informants mentioned several concerns at the individual level that may prevent employees in SMEs from participating in mental health interventions at work. In particular, worries about confidentiality, discretion and mistrust, fear of stigma, discrimination and prejudices, as well as concerns about career progression and job security (e.g., job loss, loss of status, etc.) may be a deterrent [5]. Also, employees not being interested in mental health interventions is a reported barrier. The review of McCoy et al. [30] also reported privacy concerns such as stigmatisation of high-risk groups and discriminatory job dismissal. Benning et al. [3] similarly refer to the fear that employees are not taken seriously or will be stigmatized when it comes to psychosocial risks.

Our informants highlighted methods that could be used to overcome these barriers. Promising strategies to get the buy-in from managers for mental health interventions are providing information about the economic and social benefits of workplace mental health promotion, sharing positive testimonials from other managers, and promoting the use of interventions requiring minimal time investment for managers as well as employees. Once there is buy-in of employers, information, tools and advice on how to create mentally healthy working conditions, how to establish policies about creating mentally healthy workplaces, how to deal with work stress and burnout, and how to detect and handle mental health problems among employees are, according to our informants, highly needed. The informants also highlighted the importance of putting effort into strengthening the commitment of supervisors and managers along the way by arranging regular communications about mental health. A study of Dawkins and colleagues [13] similarly emphasized the need to strengthen SME managers’ interest in engaging in mental health programs by presenting a strong business case focusing on the benefits of the program for managers, employees, and the overall business and by stipulating that such interventions are effective and worth their time and money. Although numerous studies are available on the positive effects of mental health interventions in large companies, such studies are almost non-existent in SMEs despite SMEs employing a large proportion of working populations [17, 30, 44].

Methods for encouraging employees to participate in mental health interventions, according to our informants, are: sparking their interest through campaigns, strengthening personal relationships between employees which may help to reduce stigmatizing attitudes, and increasing employees’ knowledge on mental health. Public Health England [40] additionally highlighted the importance of good relationships between leaders and their employees to improve health and wellbeing in the workplace.

One important concern was raised regarding the acceptability of online mental health tools. Despite the practical benefits of accessing such interventions via work computer equipment, two-thirds of the informants believed that employees may feel uncomfortable to do so in the workplace setting. Our results also show that employees have easier access to online interventions via their personal smartphone than via a computer [8] also report that for people working in open-plan offices, access to such interventions during work-hours is considered less feasible. Moreover, although online interventions are considered convenient and flexible to use, many employees do not have time to engage in such interventions during working hours and favour a temporary and spatial separation of work and individual web-based psychological interventions [8].

Finally, as stated [28, 32], gender is an important determinant of both mental health and employment and this consideration is also reflected in our findings as many of the informants concurred that there is a significant difference between men and women in terms of seeking help for mental health problems, with women being more likely to seek help. Women are at greater risk of suffering from depressive and anxiety disorders than men [18], which may partially be due to women being at increased risk of trauma and exposure to psychosocial risks such as sexual harassment in the workplace [7], which has been shown to nearly triple the risk of depression [14]. Furthermore, women tend to have lower status job roles and female-dominated industries are characterised by low pay and lower benefits [20]. However, a study found that employed men found it more difficult to access mental health services than employed women [31], while a study of [10] reports that the reluctance of men to seek help and to disclose about vulnerability is more pronounced in rural than in urban settings. Therefore, the differences in help-seeking may reflect partially a lower prevalence of some mental health conditions in men, as well as a tendency for some males to face additional barriers to help-seeking. The aforementioned tendencies for men and women are most likely not related to differences due to biological sex but instead to differences in gender roles: for example, a recent study in psychiatric nurses found that individual gender roles were associated with sex-specific health trajectories, including the masculine gender-role having a protective effect on trauma symptoms [26]. The same study also focused on occupational gender-roles, which may vary across different workplace settings. Many of our informants mentioned that gender-specific needs should be considered when addressing male-dominated and female-dominated workplaces. Seaton et al. [45] also mention that developing mental health interventions tailored to the specific needs of men working in a masculine workplace culture (e.g. tackling masculine ideals such as self-reliance and stoicism), are preferable in male-dominated working cultures over interventions targeting the general public. However, it must be noted that women working in male-dominated industries face particular challenges and are at risk of increased depressive symptoms [49]. Indeed, a study found that working in a sector where one’s own gender was predominant was associated with better mental health for both genders [49], highlighting the importance of not overlooking the needs of women when planning interventions for male-dominated industries.

### Strengths and limitations

A major strength of this study is that it contributes to the limited amount of scientific knowledge that is available about the needs and barriers of SMEs across Europe to become more active in implementing mental health interventions. The findings are valuable in shaping the MENTUPP intervention [1] and in giving direction to future research. Moreover, the study is comprehensive for two reasons. First, the data relate to a variety of countries in different geographical, political, cultural and economic regions, and involve a range of experts. Despite these differences, answers were largely consistent between country and expert groups. Second, the content of the survey is extensive as it addresses various research questions and the development of the survey relied on the identification of knowledge gaps in the literature and the consensus of a large number of international researchers involved in the MENTUPP consortium.

Despite these strengths, there are also some methodological limitations that need to be acknowledged. First, the study used a combination of sampling methods (networking, snowballing, internet searches) to locate key informants with specific expertise in mental health in construction, healthcare and ICT, which resulted in an unequal number of informants per country, with Albania and Hungary both being represented by ten or more informants and Australia and Germany being represented by less than five informants each. Thus, although the data were collected in several countries and comprise three different sectors of activity, they can neither at a national level nor at a sectorial level be interpreted as representative. The data in Supplementary Table 1 shows that the countries with significantly lower response rates (Australia and Germany) are countries where less than half the workforce are employed by SMEs, meaning there is the possibility that there may be some specificities to countries where a lower proportion of the population is employed in SMEs which are not captured by this survey. Second, the response rate of 44.5% was quite low, resulting in a relatively low number of informants participating in the consultation survey, although this response rate appears to be typical of web-based expert surveys [9, 11]. The response rate and variation between countries, appeared to be related to several factors. Research officers found it especially difficult to engage with experts from outside their personal network, while experts who declined to participate cited lack of time, or that they received too many requests to take part in this type of activity. A small number of experts in Germany also complained of technical issues with the German-language version of the survey. While the response rate may have biased our responses, the general trends of our results have also been found in other studies – therefore we believe that the informants responded in line with earlier findings. Third, our data did not allow us to look more in depth to sector specific results, as most participants reported to have knowledge of more than one sector or did cross-sectoral work and thus did not relate to one specific sector, as is the case for academics or representatives of occupational health association groups. Hence, our findings need to be interpreted across the three target sectors. Finally, there is a small possibility that the quality of the qualitative data is affected by translation issues as the majority of the informants were not native English speakers. However, this was mitigated by them either being fluent enough in English to directly answer in English, or the local language answers were translated by a research officer with specialist knowledge in the field. Furthermore, the thematic analysis identified consistent themes across responses from both English-speaking and other countries, and the core findings of the qualitative data largely corroborate previous study findings.

## Conclusions and future directions

In sum, our results confirm that there is a need to support SMEs more thoroughly in implementing measures to promote wellbeing and prevent non-clinical mental health issues in their employees. However, prior to implementing such interventions, employers need to be convinced that investing in employee mental health in the workplace is beneficial and thus is worth their time and money. This can be achieved by providing information about the economic and social benefits of mental health interventions and by sharing successful testimonials from other managers. Once there is buy-in from employers, their knowledge about mental health policies and interventions and competencies on how to implement mental health interventions should be increased by providing practical information. Along the way, the commitment of supervisors and managers should be strengthened and the privacy concerns of employees need to be addressed to guarantee successful implementation of interventions. Developing interventions in an online format that are accessible via smartphones may be particularly feasible as people increasingly turn to the internet to search for health care information. In male-dominated work cultures, there is evidence to support mental health interventions tailored to the specific needs and preferences of men, but it is important to ensure the specific needs of women in those industries are also met. Finally, future research is needed on the effectiveness of mental health interventions in SMEs, a gap that the MENTUPP project will help fill.

### Supplementary Information

**Additional file 1. **

## Data Availability

The dataset used and/or analysed during the current study is available from the corresponding author on reasonable request.

## References

[1] Arensman E, O’Connor C, Leduc C, Griffin E, Cully G, Ní Dhálaigh D, et al. Mental Health Promotion and Intervention in Occupational Settings: Protocol for a Pilot Study of the MENTUPP Intervention. Int J Environ Res Public Health. 2022 : 10.3390/ijerph19020947. 10.3390/ijerph19020947 PMC8775639 35055773

[2] Barros C, Baylina P, Fernandes R, Ramalho S, Arezes P (2022). Healthcare workers' mental health in pandemic times: the predict role of psychosocial risks. Saf Health Work.

[3] Benning FE, van Oostrom SH, van Nassau F, Schaap R, Anema JR, Proper KI. The Implementation of preventive health measures in small- and medium-sized enterprises – a combined quantitative/qualitative study of its determinants from the perspective of enterprise representatives. Int J Environ Res Public Health. 2022. 10.3390/ijerph19073904. 10.3390/ijerph19073904 PMC8997761 35409587

[4] Björk JM, Nordmyr J, Forsman AK. Reconciling work and family demands and related psychosocial risk and support factors among working families: a Finnish national survey study. Int J Environ Res Public Health. 2022 : 10.3390/ijerph19148566. 10.3390/ijerph19148566 PMC9318108 35886417

[5] Boren JP & Veksler AE. Communicatively Restricted Organizational Stress (CROS) I: Conceptualization and overview. Managem Commun Q. 2015, : 10.1177/0893318914558744.

[6] Braun V, Clarke V. Can I use TA? Should I use TA? Should I not use TA? Comparing reflexive thematic analysis and other pattern‐based qualitative analytic approaches. Counselling and Psychother Res. 2020. 10.1002/capr.12360.

[7] Carey KB, Norris AL, Durney SE, Shepardson RL, Carey MP. Mental health consequences of sexual assault among first-year college women. J Am Coll Health. 2018. 10.1080/07448481.2018.1431915. 10.1080/07448481.2018.1431915 PMC6311089 29405862

[8] Carolan S, de Visser RO. Employees’ perspectives on the facilitators and barriers to engaging with digital mental health interventions in the workplace: qualitative study. JMIR Ment Health. 2018. 10.2196/mental.9146. 10.2196/mental.9146 PMC5797290 29351900

[9] Chandra PS, Saraf G, Bajaj A, Satyanarayana VA. The current status of gender-sensitive mental health services for women: findings from a global survey of experts. Arch Womens Ment Health. 2019. 10.1007/s00737-019-01001-2. 10.1007/s00737-019-01001-2 31529275

[10] Coen SE, Oliffe JL, Johnson JL, Kelly MT. Looking for Mr. PG: Masculinities and men’s depression in a northern resource-based Canadian community. Health Place. 2013. 10.1016/j.healthplace.2013.01.011. 10.1016/j.healthplace.2013.01.011 23454665

[11] Cunningham CT, Quan H, Hemmelgarn B, Noseworthy T, Beck CA, Dixon E, et al. Exploring physician specialist response rates to web-based surveys. BMC Med Res Methodol. 2015. 10.1186/s12874-015-0016-z. 10.1186/s12874-015-0016-z PMC4404667 25888346

[12] Davenport LJ, Allisey AF, Page KM, LaMontagne AD, Reavley NJ. How can organisations help employees thrive? The development of guidelines for promoting positive mental health at work. Int J Workplace Health Manag. 2016. 10.1108/IJWHM-01-2016-0001.

[13] Dawkins S, Martin A, Kilpatrick M, Scott J. Reasons for engagement: SME owner-manager motivations for engaging in a workplace mental health and wellbeing intervention. J Occup Environ Med. 2018. 10.1097/JOM.0000000000001360. 10.1097/JOM.0000000000001360 29851735

[14] Diez-Canseco F, Toyama M, Hidalgo-Padilla L, Bird VJ. Systematic Review of Policies and Interventions to Prevent Sexual Harassment in the Workplace in Order to Prevent Depression. Int J Environ Res Public Health. 2022. 10.3390/ijerph192013278. 10.3390/ijerph192013278 PMC9603480 36293858

[15] Dragano N, Lunau T. Technostress at work and mental health: concepts and research results. Curr Opin Psychiatry. 2020. 10.1097/YCO.0000000000000613. 10.1097/YCO.0000000000000613 32324623

[16] Edu-Valsania S, Laguia A, Moriano JA. Burnout: A Review of Theory and Measurement. Int J Environ Res Public Health. 2022. 10.3390/ijerph19031780. 10.3390/ijerph19031780 PMC8834764 35162802

[17] Fan D, Zhu CJ, Timming AR, Su Y, Huang X, Lu Y. Using the past to map out the future of occupational health and safety research: where do we go from here? The International Journal of Human Resource Management. 2019. 10.1080/09585192.2019.1657167.

[18] Farhane-Medina NZ, Luque B, Tabernero C, Castillo-Mayén R. Factors associated with gender and sex differences in anxiety prevalence and comorbidity: a systematic review. Sci Prog. 2022. 10.1177/00368504221135469. 10.1177/00368504221135469 PMC10450496 36373774

[19] Henderson C, Evans-Lacko S, Thornicroft G. Mental illness stigma, help seeking, and public health programs. Am J Public Health. 2013. 10.2105/AJPH.2012.301056. 10.2105/AJPH.2012.301056 PMC3698814 23488489

[20] Hodges, L. Do female occupations pay less but offer more benefits? Gend Soc. 2020. 10.1177/0891243220913527.

[21] Hogg B, Medina JC, Gardoki-Souto I, Serbanescu I, Moreno-Alcázar A, Cerga-Pashoja A, et al. Workplace interventions to reduce depression and anxiety in small and medium-sized enterprises: A systematic review. J Affect Disord. 2021. 10.1016/j.jad.2021.04.071. 10.1016/j.jad.2021.04.071 34082284

[22] Hogg B, Moreno-Alcazar A, Toth MD, Serbanescu I, Aust B, Leduc C, et al. Supporting employees with mental illness and reducing mental illness-related stigma in the workplace: an expert survey. Eur Arch Psychiatry Clin Neurosci. 2022. 10.1007/s00406-022-01443-3. 10.1007/s00406-022-01443-3 PMC9305029 35867155

[23] International Classification of Diseases, Eleventh Revision (ICD-11), World Health Organization (WHO) 2019/2021. https://icd.who.int/browse11.

[24] La Montagne AD, Shann C, Martin A. Developing an integrated approach to workplace mental health: a hypothetical conversation with a small business owner. Ann Work Expo Health. 2018. 10.1093/annweh/wxy039. 10.1093/annweh/wxy039 30212883

[25] Lanaerts K, Waeyaert W, Smits I, Hauben H. Digital platform work and occupational safety and health: a policy brief. 2021. https://osha.europa.eu/en/publications/digital-platform-workand-occupational-safety-and-health-policy-brief. Accessed 13 July 2022.

[26] Kerr P, Barbosa Da Torre M, Giguere CE, Lupien SJ, Juster RP. Occupational gender roles in relation to workplace stress, allostatic load, and mental health of psychiatric hospital workers. J Psychosom Res. 2021. 10.1016/j.jpsychores.2020.110352142:110352. 10.1016/j.jpsychores.2020.110352 33450429

[27] LaMontagne AD, Martin A, Page KM, Reavley NJ, Noblet AJ, Milner AJ (2014). Workplace mental health: developing an integrated intervention approach. BMC Psychiatry.

[28] Madsen SA (2019). Men’s mental health and wellbeing: The global challenge.

[29] Maslach C, Schaufeli WB, Leiter MP (2001). Job burnout. Annu Rev Psycho.

[30] McCoy K, Stinson K, Scott K, Tenney L, Newman LS (2014). Health promotion in small business: a systematic review of factors influencing adoption and effectiveness of worksite wellness programs. J Occup Environ Med.

[31] McManus S, Bebbington P, Jenkins R, Brugha T. (eds.) (2016) Mental health and wellbeing in England: Adult Psychiatric Morbidity Survey 2014. Leeds: NHS Digital.

[32] Milner A, Scovelle AJ, King T, Marck C, McAllister A, Kavanagh A, et al. Gendered working environments as a determinant of mental health inequalities: a systematic review of 27 studies. Occup Environ Med. 2021. 10.1136/oemed-2019-106281. 10.1136/oemed-2019-106281 32817251

[33] Milner A, Witt K, LaMontagne AD, Niedhammer I. Psychosocial job stressors and suicidality: a meta-analysis and systematic review. Occup Environ Med. 2018. 10.1136/oemed-2017-104531. 10.1136/oemed-2017-104531 28851757

[34] Minas H, Jorm AF. Where there is no evidence: Use of expert consensus methods to fill the evidence gap in low-income countries and cultural minorities. Int J Ment Health Syst. 2010. 10.1186/1752-4458-4-33. 10.1186/1752-4458-4-33 PMC3016371 21176157

[35] Moll SE. The web of silence: a qualitative case study of early intervention and support for healthcare workers with mental ill-health. BMC Public Health. 2014. 10.1186/1471-2458-14-138. 10.1186/1471-2458-14-138 PMC3929153 24507543

[36] Newman LS, Stinson KE, Metcalf D, Fang H, Brockbank C, Jinnett K, et al. Implementation of a worksite wellness program targeting small businesses. J Occup Environ Med. 2015. 10.1097/JOM.0000000000000279. 10.1097/JOM.0000000000000279 PMC4274320 25563536

[37] National Institute for Occupational Safety and Health. Exposure to stress: occupational hazards in hospitals. Department of Health and Human Services, Centres for Disease Control and Prevention. 2008. 10.26616/NIOSHPUB2008136 . Accessed 14 Jan 2022.

[38] Peterson C, Sussell A, Li J, Schumacher PK, Yeoman K, Stone DM. Suicide rates by industry and occupation — national violent death reporting system, 32 States, 2016. MMWR Morb Mortal Wkly Rep. 2020. 10.15585/mmwr.mm6903a1.10.15585/mmwr.mm6903a1 PMC7367035 31971929

[39] Petrie K, Joyce S, Tan L, Henderson M, Johnson A, Nguyen H, et al. A framework to create more mentally healthy workplaces: A viewpoint. Aust N Z J Psychiatry. 2018. 10.1177/000486741772617. 10.1177/0004867417726174 28835112

[40] Public Health England (2014) Local action on health inequalities: workplace interventions to improve health and wellbeing. https://assets.publishing.service.gov.uk/government/uploads/system/uploads/attachment_data/file/356064/Review5_Employment_health_inequalities.pdf. Accessed 7 Oct 2022.

[41] Rugulies R, Aust B (2019). Work and mental health: What do we know and how can we intervene?. Scand J Work Environ Health.

[42] Salanova M, Cifre E, Llorens S, Martínez IM, Lorente L (2011). Psychosocial Risks and Positive Factors among Construction Workers.

[43] Salin D, D'Cruz P, Noronha E, Caponecchia C, Escartín J, Salin D, Tuckey M (2018). Workplace bullying and gender: an overview of empirical findings. Dignity and inclusion at work.

[44] Schreibauer EC, Hippler M, Burgess S, Rieger MA, Rind E. Work-related psychosocial stress in small and medium-sized enterprises: an integrative review. Int J Environ Res Public Health. 2020. 10.3390/ijerph17207446. 10.3390/ijerph17207446 PMC7650689 33066111

[45] Seaton CL, Bottorff JL, Jones-Bricker M, Oliffe JL, DeLeenheer D, Medhurst K. Men’s mental health promotion interventions: A scoping review. Am J Mens Health. 2017. 10.1177/1557988317728353. 10.1177/1557988317728353 PMC5675255 28884637

[46] Siegrist J, Fink G (2016). Effort-reward imbalance model. Stress: concepts, cognition, emotion, and behaviour.

[47] Siuta RL, Bergman ME (2019). Sexual harassment in the workplace. Bus Manag.

[48] Theorell T, Hammarström A, Aronsson G, Träskman Bendz L, Grape T, Hogstedt C (2015). A systematic review including meta-analysis of work environment and depressive symptoms. BMC Public Health.

[49] Tophoven S, du Prel JB, Peter R, Kretschmer V*.* Working in gender-dominated occupations and depressive symptoms: findings from the two age cohorts of the lidA study. J Labour Market Res. 2015. 10.1007/s12651-014-0165-2.

[50] Van Laar C, Meeussen L, Veldman J, Van Grootel S, Sterk N, Jacobs C. Coping with stigma in the workplace: understanding the role of threat regulation, supportive factors, and potential hidden costs. Front Psychol. 2019. 10.3389/fpsyg.2019.01879. 10.3389/fpsyg.2019.01879 PMC6718612 31507478

[51] Wagner SL, Koehn C, White MI, Harder HG, Schultz IZ, Williams-Whitt K, et al. Mental health interventions in the workplace and work outcomes: A best-evidence synthesis of systematic reviews. Int J Occup Environ Med. 2016. 10.15171/ijoem.2016.607. 10.15171/ijoem.2016.607 PMC6816521 26772593

[52] West CP, Dyrbye LN, Shanafelt TD. Physician burnout: contributors, consequences and solutions. J Intern Med. 2018. 10.1111/joim.12752. 10.1111/joim.12752 29505159

[53] World Health Organization. Mental health and substance use: mental health in the workplace. 2022. https://www.who.int/teams/mental-health-and-substance-use/mental-health-in-the-workplace. Accessed 7 Sept 2022.

