# Supplementary material for: Promoting employee wellbeing and preventing non-clinical mental health problems in the workplace: a preparatory consultation survey
Source: J Occup Med Toxicol. 2023 Aug 15;18:17. doi: 10.1186/s12995-023-00378-2 (PMC10426174; doi:10.1186/s12995-023-00378-2)
Supplement: Supplementary file 1 — Additional file 1. [file 12995_2023_378_MOESM1_ESM.docx]

**Supplementary Tables**

**Supplementary Table 1. Overview of SME and sector-specific data of each MENTUPP country**

|  | Employment data, by country | | | | | |
| --- | --- | --- | --- | --- | --- | --- |
| Country | Economy status according to World Bank | Companies which are SMEs <250 employees^a^ | Workers employed by SMEs^a^ | Workers in construction sector^b^ | Workers in healthcare sector^c^ | Workers in ICT sector^d^ |
| Albania | Upper-middle income | 99.9% | 81.4% | 9.0% | NA | NA |
| Australia | High income | 99.8% | 43.9% | 8.7% | 13.3% | NA |
| Finland | High income | 99.7% | 51.9% | 7.7% | 16.6% | 6.4% |
| Germany | High income | 99.6% | 38.8% | 5.8% | 13.4% | 3.9% |
| Hungary | Upper-middle income | 99.9% | 48.1% | 8.5% | 6.8% | 4.9% |
| Ireland | High income | 99.8% | 49.8% | 6.4% | 11.7% | 5.2% |
| Kosovo | Upper-middle income | 99.9% | 76.2% | 9.5% | NA | NA |
| The Netherlands | High income | 99.9% | 66.4% | 5.4% | 15.3% | 3.5% |
| Spain | High income | 99.9% | 62.7% | 6.7% | 7.5% | 2.7% |

Key:NA – not available or unable to source.

^a^ Sources: OECD (<https://stats.oecd.org/index.aspx?queryid=81354>); Cela M & Gaspari A (2015). Small and Medium Enterprises: Where does Albania stand? *European Scientific Journal 1;* EC Europa (<https://neighbourhood-enlargement.ec.europa.eu/system/files/2019-11/sba-fs-2019_kosovo.pdf>). Note: all sources used same definition of SME.

^b^ Sources: [Statista Research Department](https://www.statista.com/aboutus/our-research-commitment) (2023), Construction share of total employment in Europe by country. <https://www.statista.com/statistics/1302708/construction-share-of-total-employment-in-europe-by-country/> (Accessed on 20 April 2023); Back to Basics (2023) The Australian Construction Industry: Facts and Stats for 2023. https://backtobasics.edu.au/news/the-australian-construction-industry-facts-and-stats-for-2022#:~:text=As%20of%20November%202021%2C%20the,cent%20of%20the%20total%20workforce (Accessed on 20 April 2023); World Bank (2018) Job Dynamics inj Albania. https://documents1.worldbank.org/curated/en/209671528985738916/pdf/Job-dynamics-in-Albania-a-note-profiling-Albanias-labor-market.pdf (Accessed on 20 April 2023); Doroci L (2021). Market Assessment for the Construction Sector in Kosovo. https://www.researchgate.net/publication/351098649_Market_assessment_for_the_construction_sector_in_Kosovo (Accessed on 20 April 2023). Note: unable to verify consistency in definition of construction sector.

^c^ Source: OECD (2023), ICT employment (indicator). doi: 10.1787/0938c4a0-en (Accessed on 20 April 2023) OECD

^d^ Source: OECD (2023), *Health and Social Care Workforce*, in: Health at a Glance 2021. doi: 10.1787/ae3016b9-en (Accessed on 20 April 2023) OECD

**Supplementary Table 2. Overview of ESENER survey data on measures in place targeting psychosocial risks for each MENTUPP country**

|  | ESENER survey data, by % of establishments with each measure/procedure in place^a^ | | | | | | |
| --- | --- | --- | --- | --- | --- | --- | --- |
| Country | Action plans to reduce work-related stress | Information on how to include psychosocial risks in assessments | Procedures to deal with possible bullying or harassment | Measures to allow employees greater decision-making^a^ | Confidential counselling to employees | Reorganisation of work to prevent psychosocial risks | Interventions for excessively long or irregular hours |
| Albania | NA | NA | NA | NA | NA | NA | NA |
| Australia | NA | NA | NA | NA | NA | NA | NA |
| Finland | 55% | 63% | 88% | 91% | 74% | 58% | 41% |
| Germany | 25% | 59% | 37% | 68% | 53% | 52% | 47% |
| Hungary | 19% | 57% | 10% | 65% | 50% | 48% | 19% |
| Ireland | 57% | 54% | 94% | 80% | 34% | 52% | 45% |
| Kosovo | NA | NA | NA | NA | NA | NA | NA |
| The Netherlands | 38% | 68% | 36% | 75% | 39% | 36% | 19% |
| Spain | 37% | 58% | 53% | 75% | 35% | 43% | 24% |

Key: ESENER ; European Survey of Enterprises on New and Emerging Risks; NA – not available.

^a^Source: ESENER (https://osha.europa.eu/en/publications/esener-2019-overview-report-how-european-workplaces-manage-safety-and-health)
